# Fluoroscopic and gait analyses for the assessment of the functional performance of an original total ankle replacement

**DOI:** 10.1186/1757-1146-5-S1-O34

**Published:** 2012-04-10

**Authors:** Francesco Cenni, Sandro Giannini, Claudio Belvedere, Matteo Romagnoli, Lisa Berti, Maddalena Pieri, Alberto Leardini

**Affiliations:** 1 Movement Analysis Laboratory, Istituto Ortopedico Rizzoli, 40136 Bologna, Italy; 2 Department of Orthopedic Surgery, Istituto Ortopedico Rizzoli , 40136 Bologna, Italy

## Background

An original total ankle replacement design was developed with the aim of establishing compatibility between the prosthetic articulating surfaces and the retained ligaments. This was achieved with a special shape of a conforming meniscal bearing, free to move forwards/backwards on both metal components during dorsi/plantar flexion. Careful kinematics analyses were carried out in patients after this replacement to assess the functional performance during activity of daily living. A thorough assessment shall include standard gait analysis (GA) and the more accurate motion tracking of the components by 3D fluoroscopic analysis (FA).

## Materials and methods

Eleven patients implanted with the BOX Ankle (Finsbury Orthopaedics, Leatherhead-Surrey, UK) were analyzed at 12 months after surgery. GA was performed during stair-climbing/descending using a 8-cameras motion system (Vicon Motion Systems, Oxford, UK), electromyography (ZeroWire, Aurion, Milan, Italy), and an established protocol for lower limb joint kinematics and kinetics [1]. For the same patients and motor tasks, FA was performed on the same day using a standard fluoroscope (CAT Medical System, Italy) at 10Hz and an established technique [2], which works out motion of the three components in the three anatomical planes.

## Results

Nearly physiological joint kinematic patterns were observed in both legs (Table 1). A statistically significant difference between the operated and controlateral sides were found only in the hip and ankle range of flexion, and in dorsi/plantar flexion at foot strike (p<0.05). From FA, over all patients, 1.2 and 3.4 mm of antero-posterior meniscal-to-tibial translation were coupled with 5.2° and 8.2° flexion between the two metal components, respectively during stair climbing and descending. At the replaced joint, a significant correlation was found between meniscal-motion from FA and both range of flexion and flexion at foot-strike from GA.

**Table 1:** Gait analysis results

	Unit	Stair climbing		Stair descending	
		
		*Operated side*	*Controlateral side*	*Operated side*	*Controlateral side*
Hip range of flex- extension	[Deg]	59.2±4.1	46.8±6.5	24.1±5.5	16.3±4.1
Max hip flexion moment	[% BW*h]	7.5±1.2	6.3±1.1	3.2±1.2	5.9±6.7
Knee range of flexion	[Deg]	56.4±6.4	56.5±6.7	79.6±5.7	76.4±5.0
Max knee flexion moment	[% BW*h]	1.9±1.2	5.1±2.6	5.9±1.6	7.7±1.6
Ankle range of flexion	[Deg]	16.8±9.5	38.8±9.8	17.8±6.7	55.5±7.5
Ankle dorsi/plantar flexion at foot strike	[Deg]	5.9±4.3	10.9±6.6	-9.1±5.4	-27.0±6.8
Max ankle dorsi-flexion moment	[% BW*h]	6.8±1.2	7.6±1.3	6.6±1.4	7.8±1.5

## Conclusions

Nearly normal kinematics and kinetics at the main joints were observed also at the replaced leg. In addition, nearly natural function was restored at the replaced ankle, with large coupled motion in the three anatomical planes. The meniscal-motion was coupled with ankle flexion, supporting the main claims of the designers.
